# Safety and efficacy of a modified endoscopic full-thickness resection technique for gastric submucosal tumors: a case series

**DOI:** 10.3389/fonc.2024.1403517

**Published:** 2024-07-09

**Authors:** Yingsheng Zhou, Yan Huang, Wen Cheng, Jiamin Wang, Xiaoqiang Liu, Huan Peng, Juan Zhang, Qiaoqun Feng

**Affiliations:** Department of Gastroenterology, Shenzhen Guangming District People’s Hospital, Shenzhen, Guangdong, China

**Keywords:** modified endoscopic full-thickness resection, gastric submucosal tumor, precise marking and incision, predevascularization, tension-relieving closure

## Abstract

**Objectives:**

Endoscopic full-thickness resection (EFTR) has proven effective and economical for patients with gastric submucosal tumors (SMTs). However, the poor operative field of view, the risk of massive hemorrhage, and the difficulties in defect closure have limited its widespread application. Herein, we described a modified EFTR technique developed to simplify the dissection and defect closure procedures using common and economical endoscopic accessories.

**Methods:**

Forty-two patients who underwent the modified EFTR for gastric SMTs in the Shenzhen Guangming District People’s Hospital were enrolled in the case series. Following a cross incision to expose the intraluminal surface the tumors were captured by suction through a transparent cap and the roots were ligated using a loop. The tumors and part of the suction tissue were removed along the ligated root. A tension-relieving closure was performed by clipping the raised plica in four quadrants outside the ligated root. Patient demographics, tumor characteristics, and therapeutic outcomes were evaluated retrospectively.

**Results:**

All tumors had an R0 resection. The median procedure time was 51.8 min (IQR 34.25 min). No severe perioperative adverse events occurred. No residual lesion or recurrence was reported during the follow-up period of 9.84 months (IQR 5.0 months).

**Conclusion:**

The safety and practicability of Modified-EFTR could allow for wide clinical application in patients with micro-gastric SMTs.

## 1 Introduction

Gastric submucosal tumors (SMTs) are common neoplasms observed during upper gastrointestinal endoscopy, with an incidence rate of approximately 0.36% (1). While most SMTs are benign, up to 13% have malignant potential, particularly those originating from the muscularis propria layer (2). Small gastric SMTs (less than 2 cm in diameter), including “mini-SMTs” (1–2 cm) and “micro-SMTs” (<1 cm), are often detected through gastroscopy screening and primarily occur in the upper part of the stomach, especially near the gastroesophageal junction. Gastrointestinal stromal tumors (GIST) account for almost half of small gastric SMTs, followed by leiomyomas (3). These small gastric SMTs are generally regarded as having a low or very low risk of malignancy, and conservative follow-up or endoscopic resection (ER) is a common treatment approach (4). Indeed, ER has become a widely accepted option to avoid the psychological stress and economic burden of long-term follow-up along with the risk of delaying the optimal timing for treatment (5).

The premise of ER for SMTs is the absence of metastasis, and the goal is complete resection without major morbidity. For SMTs originating from the muscularis propria layer and potentially closely adhering to the serosal layer, endoscopic full-thickness resection (EFTR) is preferred under endoscopy (1). However, EFTR intentionally perforates and removes the entire tumor within the gastric wall, which may damage the rich vascular network outside the serosa layer. This can significantly increase the risk of massive hemorrhage over other ER techniques (6). In addition, sufficient exposure to the surgical field is required during operation given the high risk that the tumor will fall into the peritoneal cavity. Effective closure of defects during subsequent tumor resection is crucial to avoid peritonitis, pneumoperitoneum, and delayed perforation (7). While EFTR achieves a high en bloc resection rate for gastric SMTs, the technical difficulty and risk of complications associated with this technique restrict its widespread use. In recent years, some incision closure devices have been developed to simplify the closure such as Over-Stitch system and the over-the-scope clip (OTSC) (8, 9). Non-exposed EFTR techniques have also been designed to prevent massive hemorrhage and perforation by removing the isolated tumor above preclosed serosa using special ligation apparatuses such as the full-thickness resection device (FTRD) and the GERDX device (10). However, these devices are expensive and inaccessible, limiting their application in small- to-medium sized endoscopy centers.

In this study, we modified the conventional EFTR procedure using accessible and economical endoscopic instruments, simplifying and optimizing the procedures, making the operation more efficient and reducing the risk of complications. The aim of this study was to evaluate the safety and feasibility of modified EFTR for micro-SMTs.

## 2 Methods

### 2.1 Population

In this retrospective case series, 42 adult patients who underwent modified full-thickness resection for gastric micro-SMTs at the Digestive Endoscopy Center of Shenzhen Guangming District People’s Hospital were enrolled from January 2021 to March 2024. The inclusion criteria were as follows: 1. Patients who were 18–65 years of age had undergone modified full-thickness resection of gastric micro-SMTs. 2. Postoperative follow-up completed on schedule. The exclusion criterion was loss of follow-up. Demographic and clinical data were retrospectively obtained from electronic patient files. This study was approved by the Ethics Committee of the hospital in accordance with the principles of the Declaration of Helsinki (Brazil, 2013).

### 2.2 Endoscopic equipment and accessories

Standard single-accessory-channel endoscope (GIF-Q260J; Olympus, Tokyo, Japan) equipped with a transparent cap (MH-594; Olympus) and a high-frequency generator (VIO 200D; ERBE, Tubingen, Germany) were used in the endoscopic procedures. A carbon dioxide insufflator (UCR; Olympus) was employed to achieve carbon dioxide insufflation. Other equipment and accessories included injection needles (ATE-ZSZ-23X2000X25X4; Atetec, Jiangsu Province, China), insulation-tip knives (AMH-EK-O-2.4X1800^4^-N; Anrei, Zhejiang Province, China), disposable electric snare (MTN-PFS-E-15/23; Micro-Tech, Nanjing, Jiangsu Province, China), loop (MAJ-340; Olympus), and clips (ROCC-D-26–195; Micro-Tech, Nanjing).

### 2.3 Procedure

**Figure 1:**
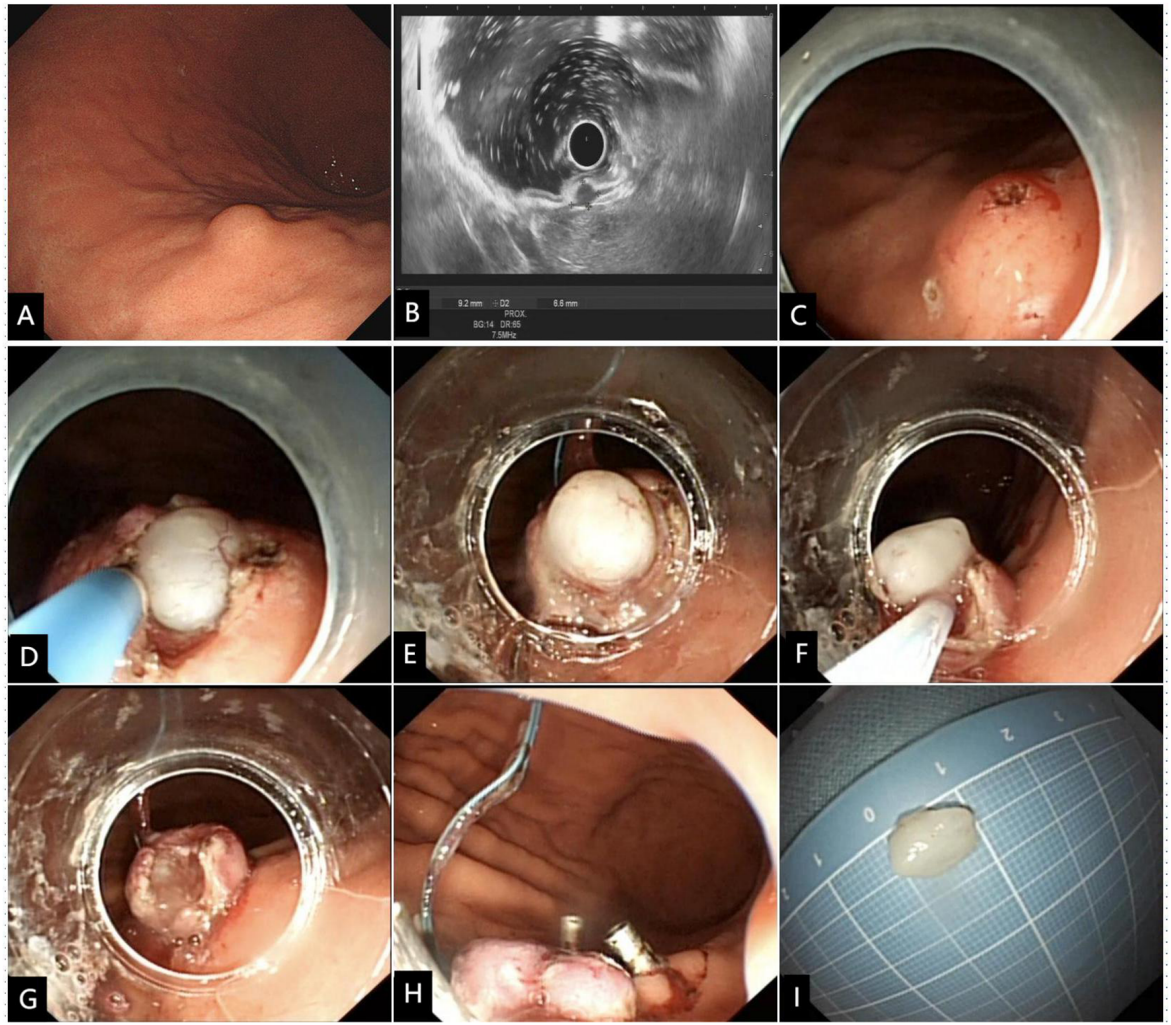
Modified full-thickness resection procedure for a representative SMT of the gastric corpus. **(A)** The SMT was initially detected by endoscopy, and **(B)** confirmed by EUS to be originated from the muscularis propria layer. **(C)** The four quadrants and apex were marked, and a small amount of submucosal water pad was injected below the five markers. **(D)** A cross incision at the center of the top mark along the four-quadrant mark point was made to expose the intraluminal surface of the tumor. **(E)** The transparent cap was used to draw out the tumor and the root was ligated with a nylon rope. **(F)** The ligated root was cut off with an electric snare to achieve a full-thickness resection. **(G)** Despite the presence of gastric wall defect, there was no bleeding or perforation of the incision. **(H)** The four-quadrant plica around the ligation point was clipped to achieve tension-relieving closure. **(I)** Macroscopic image of the resected tumor.

Preoperative endoscopic ultrasonography (EUS) was performed to determine the size, echo, growth pattern, and origin of SMTs (**Figures 1A, B**), and contrast-enhanced CT was performed to rule out metastases. The patients were placed in the left lateral decubitus position and endoscopic procedures were performed under intravenous anesthesia.

**Figure 2:**
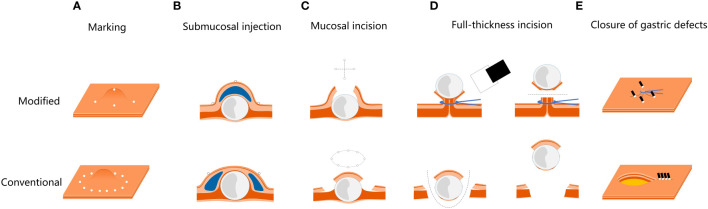
Modified EFTR procedure images, compared with the conventional EFTR. **(A)** The modified marker is more accurate for tumor location. **(B, C)** The procedure of submucosal injection and mucosal incision are modified, making the incision smaller and less prone to perforation. **(D)** Predevascularization and closure of serosa are performed to prevent bleeding and perforation during full-thickness resection. **(E)** Based on predevascularization, tension-relieving closure is performed to reduce the difficulty to close the gastric defect.

Modified full-thickness resection procedure (**Figures 1**, **2**, **Supplementary Video 1**). Step 1. Precise marking. A five-point method, namely the top and four quadrants, was used to accurately locate the tumor and mark the boundaries. Based on the diameter of the tumor measured by EUS, the boundary marks were corrected in four quadrants around the top mark of the tumor (**Figures 1B, C**, **2A**). Step 2. Anti-displacement submucosal injection. A submucosal saline injection was administered, keeping the top marker at the peak of the submucosal water pad and the four-quadrant markers symmetrical distribution in the periphery of the water pad (**Figures 1C**, **2B**). Step 3. Small mucosal incision within boundary markers. The intraluminal surface of the tumor was exposed by a smaller cross incision along the four-quadrant marker centered on the top marker (**Figures 1D**, **2C**). Step 4. Predevascularization and preclosed serosa. The blood supply to the tumor was then preblocked subsequently. After the tumor and its surrounding tissue, especially the adjacent serosal tissue, were sufficiently sucked into a transparent cap, the captured lesion root was ring-ligated with a loop (**Figures 1E**, **2D**). Step 5. Full-thickness resection. The tumor and part of the suction tissue were removed along the ligated root to ensure the integrity of the tumor capsule (**Figures 1F, G**, **2D**). Step 6. Tension-relieving closure. The raised plica in four quadrants about 5 mm outside the ligation point were clipped to reduce incision tension. Clips were used to close the incision based on the condition of the gastric wall defect where the loop was ligated (**Figures 1H**, **2E**).

### 2.4 Outcome

The primary outcome variable was technical success, defined as successful tumor resection using modified EFTR. The evaluation criteria included: 1. Endoscopic en bloc resection: the resected tumor was en bloc with intact capsule. 2. Histological R0 resection: postoperative specimen pathology showed no tumor invasion at the resection margin.

Secondary outcome variables included procedure time and perioperative adverse events. Adverse events were defined as any events that interfered with the planned treatment, mainly including bleeding, perforation, and infection.

Bleeding included intraoperative and delayed bleeding. Intraoperative bleeding was classified according to bleeding volume during the operation and the impact of endoscopic operation. Grade 0: the absence of or trace intraoperative bleeding, no need for endoscopic hemostasis, and no impact on visibility during the operation. Grade 1: Intraoperative bleeding requiring endoscopic hemostasis. The operation could still be completed and the patient’s vital signs remained stable during ER. Grade 2: ER is aborted due to the failure of endoscopic hemostasis for intraoperative massive bleeding, or the patient’s vital signs are unstable due to blood loss. Delayed bleeding is defined as postoperative hematemesis, melena, fresh blood in the nasogastric tube, or a decrease of more than 20 g/L in hemoglobin level.

Postoperative perforation was defined as severe abdominal pain and peritoneal irritation after operation, and gastrointestinal perforation at the operative site was detected by abdominal computed tomography (CT) scan or endoscopy.

Postoperative infection was defined as systemic inflammatory manifestations such as fever or elevated infection indicators after operation after excluding other causes of infection.

**Figure 3:**
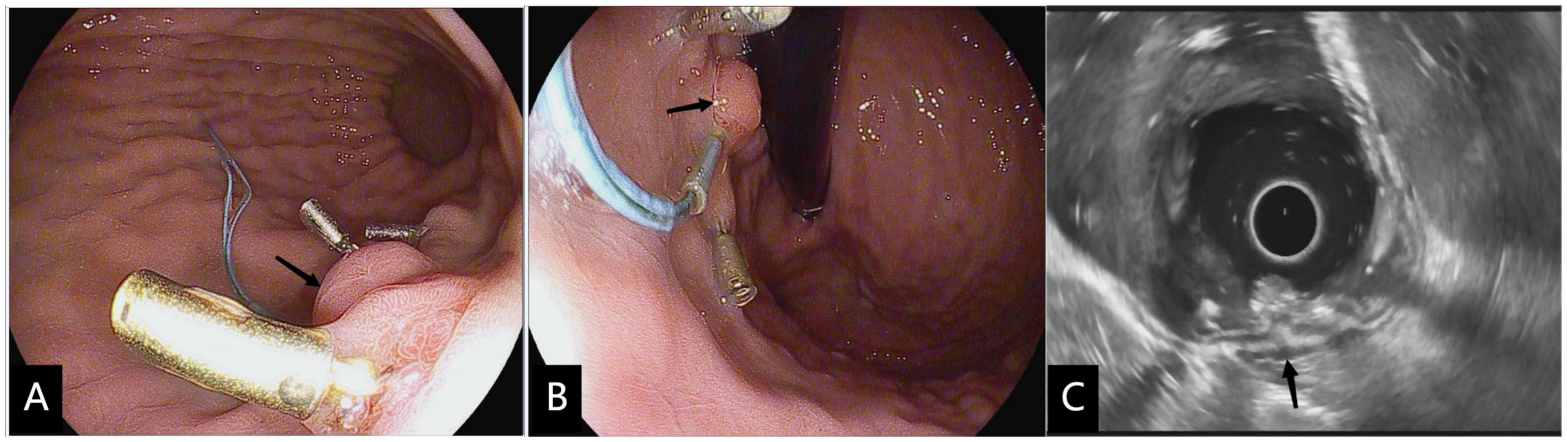
Three months after modified full-thickness resection procedure. **(A, B)** Upper gastrointestinal endoscopy performed 3 weeks after modified full-thickness resection procedure showed the ligated root hyperplastic changes(black arrow) and residual four-quadrant clips. **(C)** EUS confirmed that no hypoechoic mass residual was found in the muscularis propria at the resection site (black arrow).

Follow-up: endoscopic follow-up was performed at 1, 3, 6, and 12 months after modified EFTR (**Figure 3**), once annually thereafter. Patients diagnosed with gastrointestinal stromal tumors (GIST) underwent contrast-enhanced CT scans at a frequency that corresponded with their histological risk classification (3). The presence of residual or recurrent tumors was reconfirmed by the above examination.

### 2.5 Statistical analysis

Descriptive statistics were analysis using SPSS statistical software version 26 (IBM Corp., Armonk, New York, USA). The classification results are presented as frequencies and percentages. Continuous outcomes are presented as medians and interquartile ranges (IQRs) due to the limited study sample size.

## 3 Results

### 3.1 Baseline characteristics

Baseline characteristics and tumor features of the included patients are summarized in **Table 1**. The enrolled patients had a median age of 48.52 years (IQR 17.5 years) and 18 (42.86%) were male. All tumors originated from the muscularis propria layer, and most were located in gastric fundus (n = 26, 61.9%) or gastric corpus (n = 16, 38.1%). Most lesions grew in an intraluminal pattern (n = 36, 85.71%), and a small percentage grew in both intraluminal and extraluminal patterns (n = 6, 14.29%). The median tumor size assessed by EUS before operation was 8.31 mm (IOR 3.0 mm) in diameter.

**Table 1:** Baseline characteristics and tumor features.

	Total (n = 42)
Age, median (IQR), years	48.52 (17.5)
Male sex, n (%)	18 (42.86)
BMI, median (IQR), kg/m^2^	23.35 (5.14)
ASA score, n (%)	
0	42 (100)
1	0 (0)
Tumor location, n (%)	
Fundus	26 (61.9)
Corpus	16 (38.1)
Depth of invasion, n (%)	
Muscularis propria layer	42 (100)
Tumor growth pattern, n (%)	
Intraluminal	36 (85.71)
Intraluminal and extraluminal	6 (14.29)
Tumor size, median (IQR), mm	8.31 (3.0)

### 3.2 Technical success

Therapeutic outcomes of modified EFTR were summarized in **Table 2**. The median procedure time was 51.38 min (IQR 34.25 min). Both en bloc resection rate and histological R0 resection were 100%. The pathological diagnoses were GISTs (n = 21,50.0%) and leiomyomas (n = 21, 50.0%), respectively. All GISTs were very-low histological risk.

**Table 2:** Therapeutic outcomes.

	Total (n = 42)
Procedure time, median (IQR), min	51.38 (34.25)
En bloc resection, n (%)	42 (100)
Histological R0 resection, n (%)	42 (100)
Pathological diagnosis, n (%)	
Gastrointestinal stromal tumor	21 (50)
Leiomyoma	21 (50)
Adverse events, n (%)	
Infection	0 (0)
Intraoperative bleeding	
Grade 0	42 (100)
Delayed bleeding	0 (0)
Delayed perforation	0 (0)
Follow-up, median (IQR), months	9.84 (5.0)
Residue, n (%)	0 (0)
Recurrence, n (%)	0 (0)

### 3.3 Adverse events

No infection, delayed perforation, delayed bleeding, or other serious complications occurred. Intraoperative blood loss was graded as minimal in all patients.

### 3.4 Follow-up

During the median follow-up of 9.84 months (IQR 5.0 months), no residual tumor or recurrences were reported.

## 4 Discussion

This study described the use of a modified EFTR for micro-SMTs. This technique has the advantages of accurate localization of gastric micro-SMTs, a small incision, low incidence of bleeding and perforation, and easy tumor dissection and incision suturing. This retrospective study preliminarily demonstrated the clinical safety and efficacy of this modified EFTR. All tumors underwent R0 resection, no serious adverse events occurred after operation, and no tumor residual and recurrences occurred during follow-up.

Gastric SMT is usually asymptomatic, found incidentally during endoscopic screening, and difficult to accurately diagnose even after biopsy or EUS-FNA (11). Endoscopic treatment is an effective, safe, economical, and minimally invasive method for accurate histopathological evaluation and cure of SMTs (12). Thus, the value of endoscopic treatment of micro-SMTs during the early stages of disease should not be underestimated. EFTR evolved from ESD and is a preferred method for treating SMTs that originate from the deep muscularis propria layer. The feature of EFTR is to ensure en bloc resection of the tumor and perform iatrogenic perforation (13). Even for experienced operators, this is an extremely challenging job. These operations often cause iatrogenic perforation, greatly increasing the risk of intraoperative bleeding that causes the gastric cavity to collapse, limiting the operative field, and leading to operational difficulties. In addition, tumors growing extraluminal are at risk of falling into the peritoneal cavity during resection. However, the annular perforation and edema in the incision edge make it more difficult to close the incision. Long-time intraperitoneal exposure to gastric contents and air further increases the risk of local peritonitis and pneumoperitoneum. With the progress of endoscopic technology, the effectiveness of nonexposed EFTR in reducing the risk of bleeding and perforation has been confirmed in a number of studies, however, the paucity and maneuverability of the special equipment required, and the size and anatomic location of resectable lesions may limit their widespread applicability (10).

This study took advantage of the non-exposed EFTR presealing serosal layers and achieves this effect using affordable and easily accessible devices such as transparent caps and loops (10, 14). The transparent cap we used had a diameter of 13.9 mm and a front mounting exposed length of 5 mm. In theory, tumors within 11.2-mm in diameter can be completely attracted into the cap channel using an effective suction volume. Thus, it is feasible to use transparent cap suction combined with nylon rope ligation to completely capture <11.2 mm tumors by positioning and exposing the intraluminal surface. Closure of serosa and pre-devascularization can be easily achieved using caps and loops. Clips were then used to reduce tension around the ligated root to further reduce the risk of delayed perforation. The flexibility of these devices could make them suitable for all regions of the stomach.

Adequate tumor exposure before resection is essential for en-bloc resection, allowing more accurate and complete tumor capture in a limited space. In this study, the submucosal injection and incision steps of conventional EFTR were retained and optimized. Indeed, the R0 resection rate of modified EFTR was higher than that of non-exposed EFTR reported in prior studies (100% vs. 81.2%–87.0%) (10). Another highlight of the modified EFTR described in the current study is accurate marker localization and small incision. After conventional EFTR marker injection, micro-SMTs are prone to shift under the water pad due to their small size, requiring a complete mucosal incision outside the marker or additional incision to find the displaced tumor. Using the modified EFTR, the tumor position was vertically locked below the marker point at the center of the water pad using accurate marker positioning and injection. The tumor was also accurately exposed by a small incision through the medial incision of the peripheral marker point. In addition, the use of an insulation-tip knife to cut at the top of the tumor not only reduce the risk of perforation during mucosal incision by inexperienced operators but also reduce the risk of damage to the tumor capsule during incision. This study optimized EFTR and achieved a high technical success rate using simplified operational steps. This can be attributed to the strict compliance of indications and the highly scientific operative design.

The modified EFTR in this study greatly reduces the difficulty of operation, while preserving the effectiveness and safety of the endoscopic procedures to the greatest extent. The biggest technical difficulties of conventional EFTR are tumor dissection and incision closure. A retrospective study from different tertiary hospitals in China evaluated 35 gastric GISTs (<2 cm) who received conventional EFTR with a mean procedure time of 91 ± 63 minutes (10). Another large retrospective study in a tertiary referral center in China assessed 536 patients with upper intestinal SMTs arising from the muscularis propria. Those managed with conventional EFTR or STER had a complication rate of 12.9%, including 12.1% with perioperative perforation and 1.8% with perioperative bleeding (6). Meanwhile, the current study demonstrated a shorter procedure time (51.38 min, IQR 34.25 min) and fewer complications(0%). Since the modified operation uses a transparent cap to assist in capturing the tumor and close the serosa layer rather than conventional endoscopic dissection technology to separate the tumor, the modified endoscopic procedure is simpler and carries a lower risk of bleeding and perforation. Remarkably, modified EFTR is likely to have a shorter learning curve than conventional EFTR, which requires more ESD and submucosal endoscopic skills and experience.

This study has several limitations. First, this was a retrospective study conducted in a single endoscopic center with a small sample size. Second, the study lacked a control group. Third, the efficacy of the modified method may be limited by the diameter of the transparent cap and the suction force of the endoscope. This study is theoretically only applicable to micro-SMTs. Future research should directly compare the technical success rate and adverse events of the conventional and modified technique using multicenter, large-sample, and long-term clinical observational studies. If a large-diameter transparent cap and a precise suction device are developed in the future, it is expected to expand the indications for this modified EFTR of gastric SMTs with a diameter of more than 1 cm.

In conclusion, the modified EFTR technique described here significantly reduces the challenges of endoscopic en bloc resection of gastric micro-SMTs without a need for additional auxiliary high-value instruments and carries a lower risk of bleeding, perforation, and infection. This method should be more widely adopted and applied in clinical settings.

## Data availability statement

The original contributions presented in the study are included in the article/**Supplementary Material**. Further inquiries can be directed to the corresponding author.

## Ethics statement

The studies involving humans were approved by the ethics committee of Shenzhen Guangming District People’s Hospital. The studies were conducted in accordance with the local legislation and institutional requirements. In this study, data obtained from previous clinical diagnosis and treatment and the risk to the subjects was not greater than the minimal risk. Waiver of informed consent would not adversely affect the rights and health of the subjects. If informed consent is required, patients may have abdominal pain and other somatic symptoms during the observation of postoperative complications after knowing the change of operative methods, which will affect the credibility of postoperative observation and the study will not be conducted rigorously. The privacy and personally identifiable information of the subjects were protected. Follow-up in this study was based on the frequency and content recommended by the current clinical guidelines, and there was no need to obtain additional subject information.

## Author contributions

YZ: Conceptualization, Funding acquisition, Project administration, Validation, Writing – original draft. YH: Data curation, Formal analysis, Methodology, Software, Writing – review & editing. WC: Conceptualization, Data curation, Formal analysis, Supervision, Visualization, Writing – original draft, Writing – review & editing. JW: Data curation, Investigation, Methodology, Validation, Writing – review & editing. XL: Investigation, Methodology, Validation, Writing – review & editing. HP: Investigation, Validation, Writing – review & editing. JZ: Investigation, Validation, Writing – review & editing. QF: Investigation, Validation, Writing – review & editing.
